# Supplementary material for: SWI/SNF and the histone chaperone Rtt106 drive expression of the Pleiotropic Drug Resistance network genes
Source: Nat Commun. 2022 Apr 12;13:1968. doi: 10.1038/s41467-022-29591-z (PMC9005695; doi:10.1038/s41467-022-29591-z)
Supplement: Supplementary file 1 — Supplementary information [file 41467_2022_29591_MOESM1_ESM.pdf]

Supplementary Information

SWI/SNF and the histone chaperone Rtt106 drive expression of the Pleiotropic Drug Resistance network genes

Vladislav N. Nikolov, Dhara Malavia, Takashi Kubota

Inventory of Supplementary Information

Supplementary Figure 1
Genes whose promoters are bound by Rtt106 and ontology analysis

Supplementary Figure 2
The most differentially expressed genes in *rtt106Δ* in YPD, and testing conditions that induce expression of *PDR5*

Supplementary Figure 3
Rtt106 is required for Pdr3-dependent expression of *PDR5* during log phase, but not for ketoconazole-induced Pdr1-dependent expression of *PDR5*

Supplementary Figure 4
Construction of epitope-tagged *PDR3* and ChIP-qPCR analysis of Pdr3.

Supplementary Figure 5
Sensitivity to ketoconazole of *fzo1Δ* and mitochondrial DNA-depleted strains.

Supplementary Figure 6
Minichromosome isolation, the contribution of the SWI/SNF subunits to azole antifungal resistance and gene expression of *PDR5*, and purification of the SWI/SNF complex

Supplementary Figure 7
Nucleosome positioning and occupancy at the *PDR5* locus in the presence and absence of Snf2 and Rtt106, and the number of promoters bound by SWI/SNF and/or Rtt106.

Supplementary Figure 8
RNA-seq and ChIP-qPCR analyses of *C. glabrata* strains.

Supplementary Table 1
Changes of mRNA level of PDR genes in absence of Rtt106 and Pdr3 in YPD analysed by RNA-seq

Supplementary Table 2
Changes of mRNA levels of genes in Types A, B and C in the absence of Rtt106 and Snf2 in YPD, analysed by RNA-seq

Supplementary Table 3
Yeast strains used in this study

Supplementary References

a

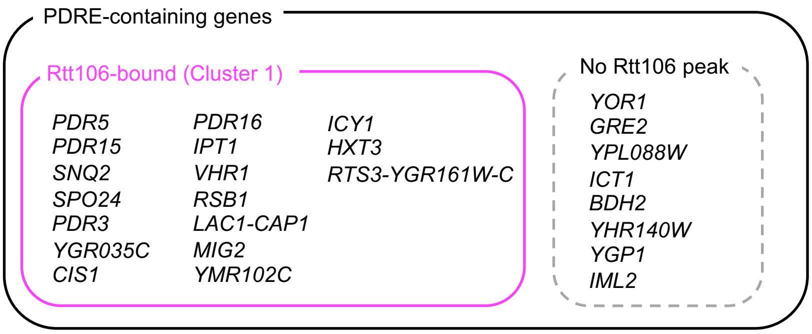

b

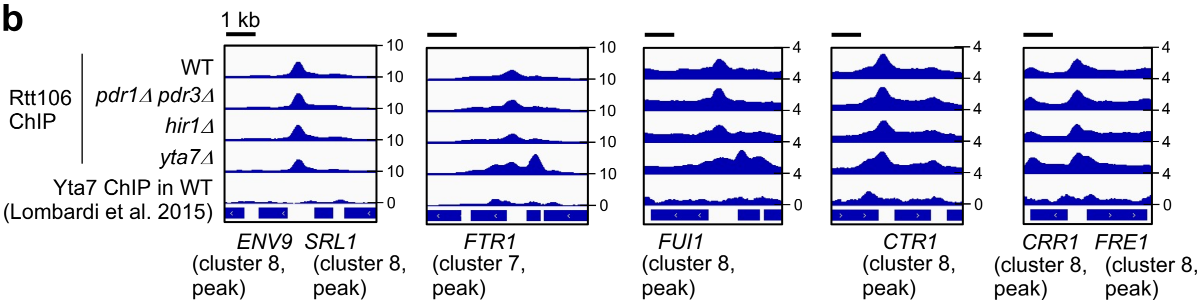

c

Gene Ontology: 'peak' in clusters 7 & 8 (=Type C), 50 genes

Gene Ontology term	Frequency	Genome frequency	P-value	Genes annotated to the term
Cell periphery	19 of 50 genes, 38.0%	846 of 7166 genes, 11.8%	0.00015	*SSA2, CWP2, SIM1, FTR1, PMA1, FET3, CRR1, PHO87, FKS1, PUT4, CTR1, FRE1, FUI1, GIC2, SUN4, SRL1, UTH1, FRE4, KCC4*
Cell surface	4 of 50 genes, 8.0%	20 of 7166 genes, 0.3%	0.00088	*SRL1, SUN4, SIM1, UTH1*
Cyclin-dependent protein kinase holoenzyme complex	4 of 50 genes, 8.0%	28 of 7166 genes, 0.4%	0.00360	*PCL2, CLB1, CLN2, PCL1*
Fungal-type cell wall	7 of 50 genes, 14.0%	136 of 7166 genes, 1.9%	0.00364	*SIM1, UTH1, CWP2, SSA2, SRL1, SUN4, CRR1*

d

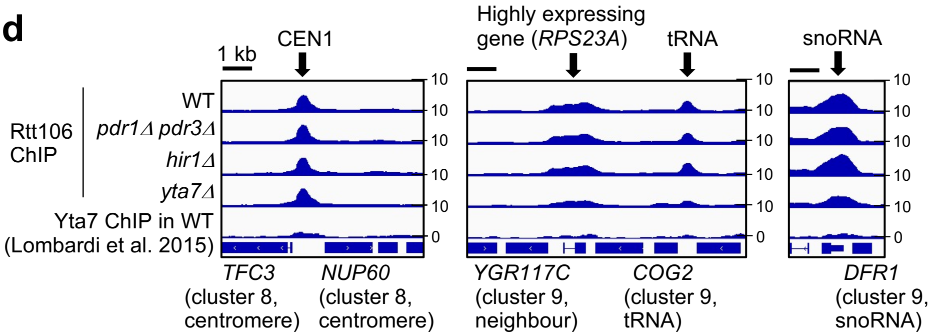

Supplementary Figure 1. Genes whose promoters are bound by Rtt106 and ontology analysis.
(a) Genes with at least one PDR responsive element (PDRE) within their promoters. Gene promoters bound by Rtt106 (Magenta, Cluster 1 as in Fig 2) and those not (dashed grey) are shown separately. (b) Specimen gene promoters categorised into 'peaks' in clusters 7 and 8 (=Type C). Yta7 binding tested by ChIP-seq analysis performed by Lombardi et al. 2015 (ref. 1) is shown alongside Rtt106 binding (c) Gene ontology analysis of the genes categorised to 'peaks' in clusters 7 and 8 (=Type C). P-values were calculated by a hypergeometric distribution with Bonferroni Correction in GO Term Finder. (d) Specimens of gene promoters containing centromere, tRNA, snoRNA or highly expressing gene. These promoters are considered as 'false positives' because Rtt106 most likely binds centromeres, tRNA, snoRNA and highly expressing genes, rather than binding to their promoters.

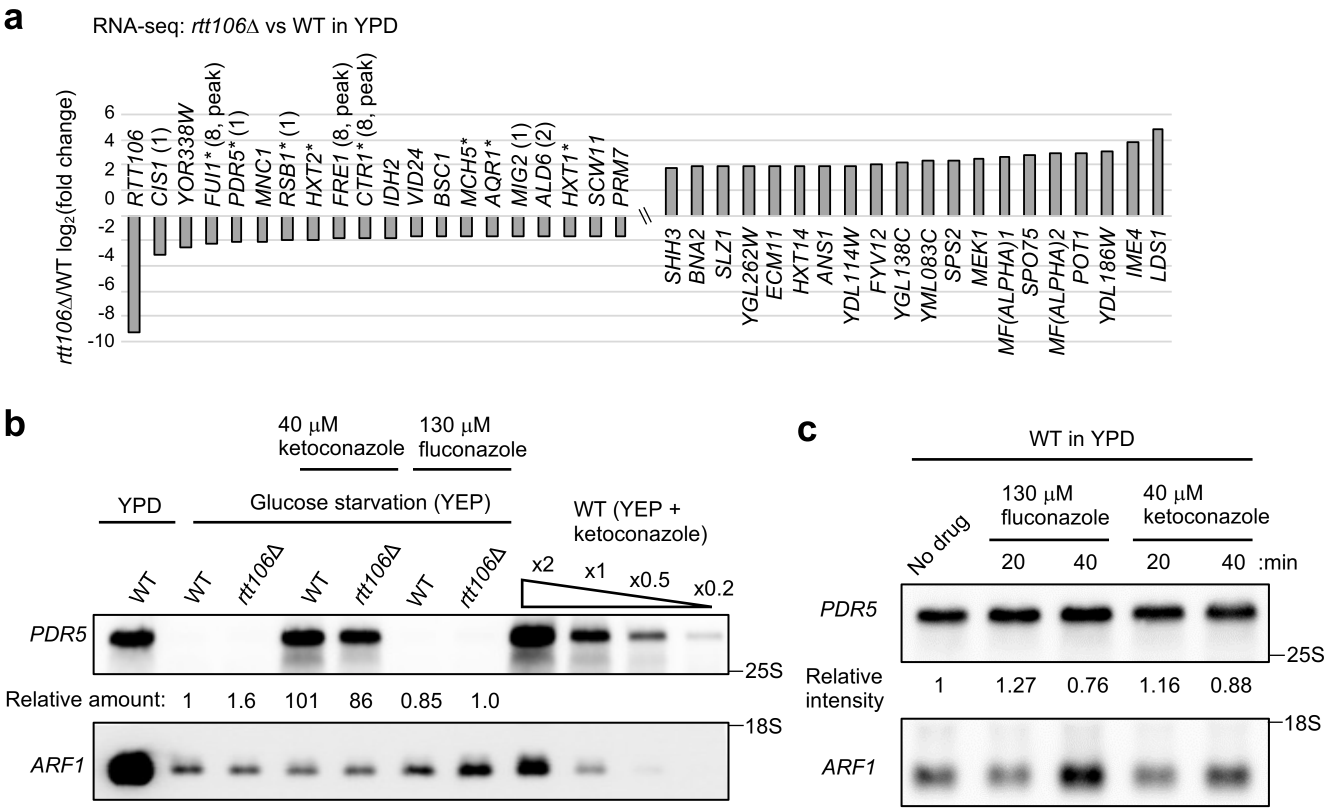

Supplementary Figure 2. The most differentially expressed genes in *rtt106*D in YPD, and testing conditions that induce expression of *PDR5*.

(a) Log2 fold changes of mRNA level of the most increased and decreased 20 genes in *rtt106*D compared to WT grown in YPD. Numbers in brackets, cluster number. Data represents means of three biological replicates. Asterisks, transporter genes. (b) Northern blot analysis of *PDR5* mRNA in WT and *rtt106Δ* treated with fluconazole and ketoconazole transiently (15 min) under glucose-starved conditions (YEP). Serial dilutions of the WT RNA sample treated with ketoconazole were loaded for quantification. The amounts of *PDR5* mRNA relative to that prepared from WT in YEP are shown below the *PDR5* blot. *ARF1*, loading control. The positions of 25S and 18S rRNAs are shown. (c) Northern blot analysis of *PDR5* mRNA in WT treated with fluconazole and ketoconazole in YPD. The intensities relative to *PDR5* mRNA prepared from WT without drug treatment are shown below the *PDR5* blot. *ARF1*, loading control. The positions of 25S and 18S rRNAs are shown.

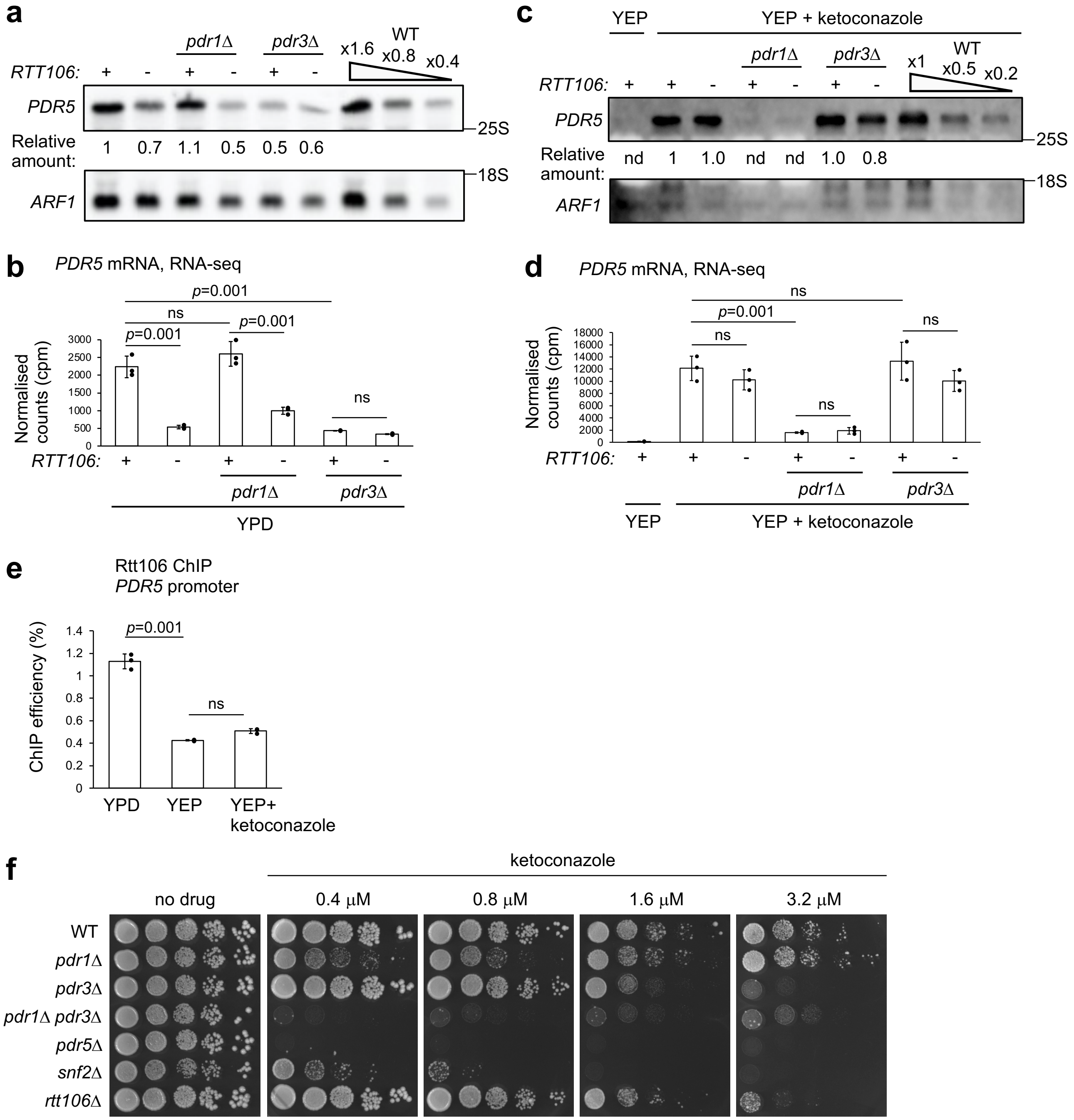

Supplementary Figure 3. Rtt106 is required for Pdr3-dependent expression of *PDR5* during log phase, but not for ketoconazole-induced Pdr1-dependent expression of *PDR5*.

(a) Northern blot analysis showing dependence of *PDR5* mRNA in WT and *rtt106*Δ on Pdr1 or Pdr3. For quantification, a serial dilution of the RNA sample prepared from WT grown in YPD was loaded. The amounts of *PDR5* mRNA relative to that prepared from WT in YPD are shown below the *PDR5* blot. *ARF1*, loading control. The positions of 25S and 18S rRNAs are shown. (b) Normalised counts of *PDR5* mRNA extracted from RNA-seq data of WT and *rtt106*Δ with and without Pdr1 and Pdr3. Data represents means of three biological replicates. Error bars, standard deviations. Significance determined by one-way ANOVA with post-hoc Tukey HSD test. ns, P>0.05. (c) Northern blot analysis showing dependence of *PDR5* mRNA in WT and *rtt106*Δ on Pdr1 or Pdr3 as in A, but treated with ketoconazole. The positions of 25S and 18S rRNAs are shown. (d) Normalised counts of *PDR5* mRNA extracted from RNA-seq data of WT and *rtt106*Δ with and without Pdr1 and Pdr3 as in panel b, but treated with ketoconazole. Data represents means of three biological replicates. Significance determined by one-way ANOVA with post-hoc Tukey HSD test. ns, P>0.05. (e) ChIP-qPCR analysis of Rtt106 in YPD, YEP and YEP plus ketoconazole to test Rtt106 binding at *PDR5* promoter. Data are presented as mean values +/- SD (n = 3 biological replicates). Significances determined by one-way ANOVA with post-hoc Tukey HSD test. ns, P>0.05. (f) Sensitivity to ketoconazole of WT, *pdr1*Δ, *pdr3*Δ, *pdr1*Δ *pdr3*Δ, *pdr5*Δ, *snf2*Δ, and *rtt106*Δ. Five-fold dilutions of indicated strains were spotted on YPD and YPD containing ketoconazole and incubated at 30°C for 3 days.

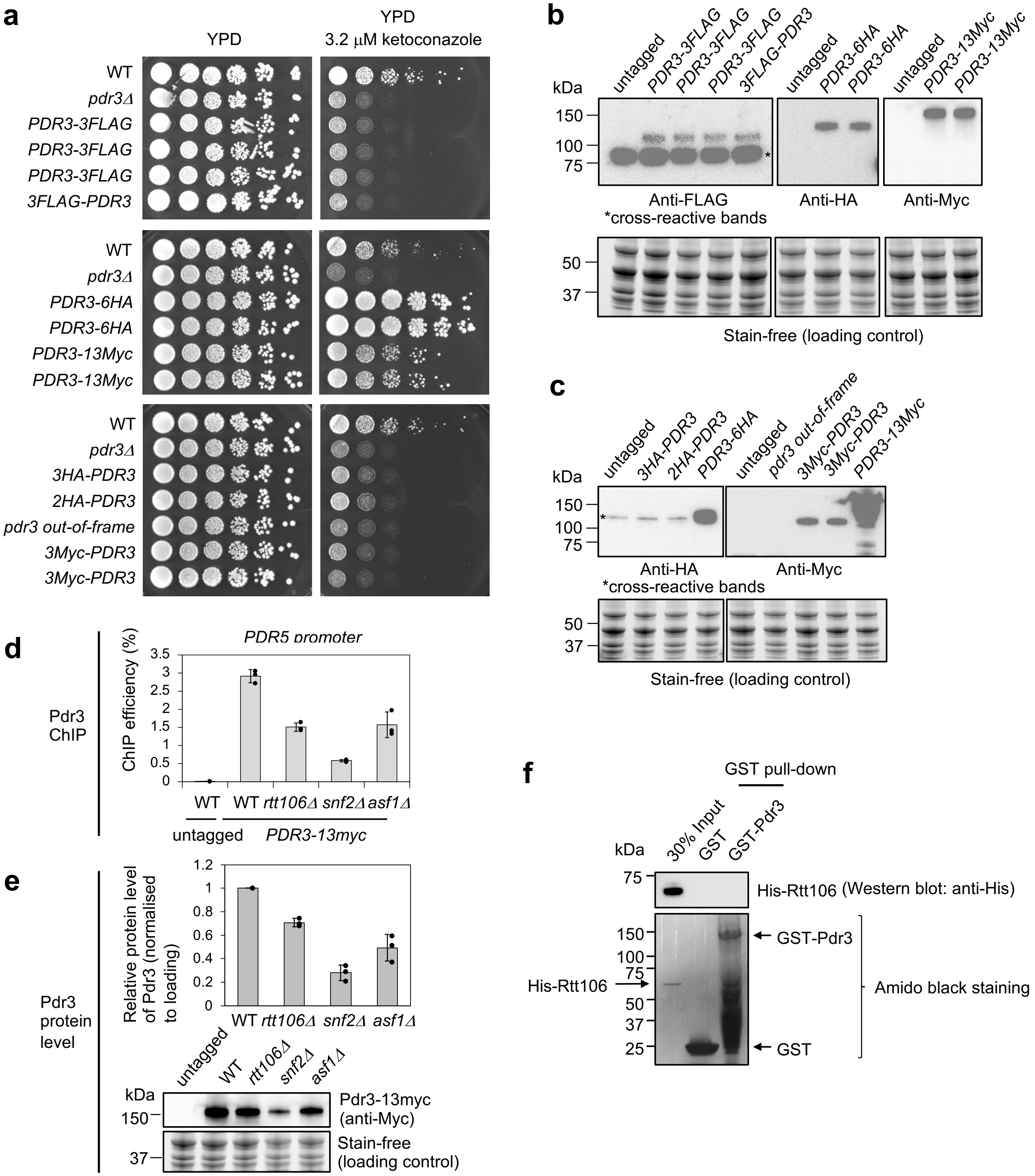

Supplementary Figure 4. Construction of epitope-tagged *PDR3* and ChIP-qPCR analysis of Pdr3.
(a) Functionality tests for a series of epitope-tagged Pdr3 by drug sensitivity assays show that *PDR3-13Myc* is the best tagged *PDR3* strain among the tags tested. Five-fold dilutions of indicated strains were spotted on YPD and YPD containing ketoconazole and incubated at 30°C for 3 days. 1-3 isolates per strain were tested. All N-terminally tagged *PDR3* strains showed were constructed by CRISPR-Cas9 genome editing. '*pdr3 out-of-frame*', a frame shift mutation introduced when 3Myc was inserted. (b and c) Expression check of the indicated strains by western blot. Expression of neither 3HA-Pdr3 nor 2HA-Pdr3 was confirmed. (d) ChIP-qPCR analyses of Pdr3-13myc at the *PDR5* promoter in the indicated strains grown in YPD. ChIP efficiency, the recovery of ChIPed DNA relative to the amount of input. Data are presented as mean values +/- SD (n = 3 biological replicates). (e) Protein levels of Pdr3 in whole cell extracts from the indicated strains grown in YPD. The histogram shows protein level of Pdr3 relative to WT (normalised to loading), represented as mean values +/- SD (n = 3 biological replicates). The panels below show a western blot and a Stain-free image of one of the three experiments. (f) GST pull-down analysis of GST-Pdr3 and His-Rtt106, both of which are purified from *E. coli*.

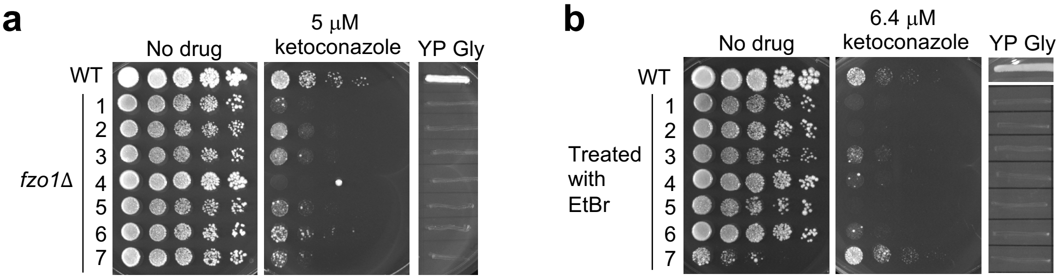

Supplementary Figure 5. Sensitivity to ketoconazole of *fzo1Δ* and mitochondrial DNA-depleted strains. (a and b) Sensitivity to ketoconazole of WT and 7 isolates of *fzo1*Δ (a) and 7 isolates of mitochondrial DNA (mtDNA)-depleted strains by ethidium bromide (EtBr) treatment (b). Five-fold dilutions of indicated strains were spotted on YPD and YPD containing ketoconazole and incubated at 30°C for 3 days. Loss of mtDNA was evaluated by growth defect in YEP containing glycerol (YP Gly). In inconsistent with previous reports, loss of mtDNA did not cause resistance to azole antifungal drug in the strain background used in this study. BY4741 is used in this study, while SEY6210 in the previous studies.

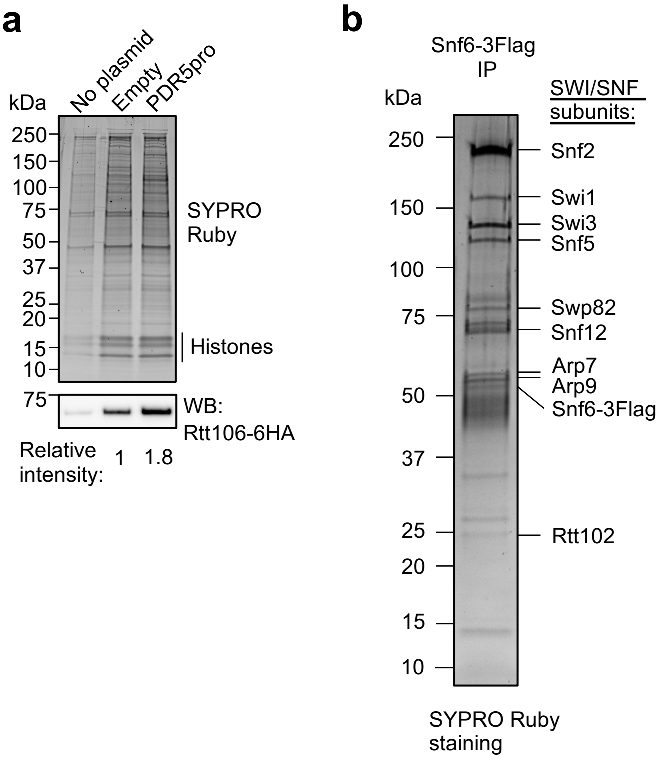

c

Protein name	# PSMs	MW [kDa]
Uba4	215	49.3
Swi1	53	147.8
Snf5	51	102.5
Mec1	45	273.2
Snf2	45	193.9
Swi3	36	92.9
Snf6	34	37.6
Snf12	32	63.8
Arp9	30	53
Arp7	29	53.8
Swp82	22	70.2
Ssa2	22	69.4
Vma2	14	57.7
Rfa3	11	13.8
Rim1	11	15.4
Tef2	10	50
Ddc2	9	86.4
Rfa2	6	29.9
Rfa1	5	70.3
Pma2	3	102.1
Rtt102	3	17.8
Act1	3	41.7
Pdr1	3	121.7
Rpl19b	3	21.7
Rpp0	3	33.7

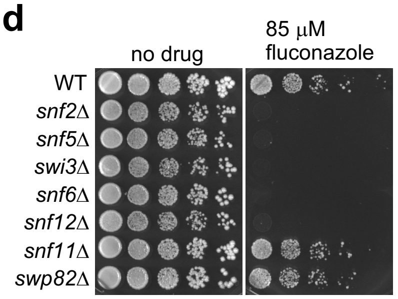

e

Kemmeren et al. 2014, 1487 mutants

			PDR5 mRNA, log2 ratio (mutant/WT)
Decreased Top 7	*pdr5*Δ	PDR5 itself	-5.2
	*snf5*Δ	SWI/SNF	-2.5
	*med15*Δ	Mediator	-2.5
	*swi3*Δ	SWI/SNF	-2.4
	*med2*Δ	Mediator	-2.3
	*spt20*Δ	SAGA	-2.3
	*snf2*Δ	SWI/SNF	-2.2
Other SWI/SNF subunits tested	*snf6*Δ	SWI/SNF	-1.7
	*snf12*Δ	SWI/SNF	-1.9
	*snf11*Δ	SWI/SNF	0.05
	*swp82*Δ	SWI/SNF	-0.08

Supplementary Figure 6. Minichromosome isolation, the contribution of the SWI/SNF subunits to azole antifungal resistance and gene expression of *PDR5*, and purification of the SWI/SNF complex. (a) Proteins remain associated with purified minichromosomes analysed by SYPRO Ruby staining and western blotting (WB), and anti-HA antibody to detect Rtt106. Minichromosome isolation was performed as in Fig. 5 and described in Methods. Relative intensity of the Rtt106 band is shown below the western blot. (b and c) The immunopurified fraction of the SWI/SNF complex (via Snf6-3FLAG) from yeast cells was analysed by SYPRO Ruby staining (b) and by mass spectrometry (c). # PSMs, the number of peptide spectrum matches. All proteins listed were identified by at least three PSMs were listed. Subunits of SWI/SNF (cyan), Replication and repair proteins (yellow), and Pdr1 (red) are highlighted. The interaction of SWI/SNF with the checkpoint kinase Mec1-Ddc2 complex (a homolog of human ATR-ATRIP) was previously reported[2]. Ddc2 binds Replication Protein A (RPA) composed of Rfa1, Rfa2, and Rfa3 (ref. 3). (d) Sensitivity to fluconazole of deletion mutants of the SWI/SNF subunits. Five-fold dilutions of indicated strains were spotted on YPD and YPD containing fluconazole and incubated at 30°C for 3 days. (e) The top 7 mutants exhibiting the most decreased level of *PDR5* mRNA, along with the mutants of other SWI/SNF subunits. Changes in *PDR5* mRNA in the indicated mutants (from 1487 mutants tested) were extracted from Kemmeren et al. 2014 (ref. 4).

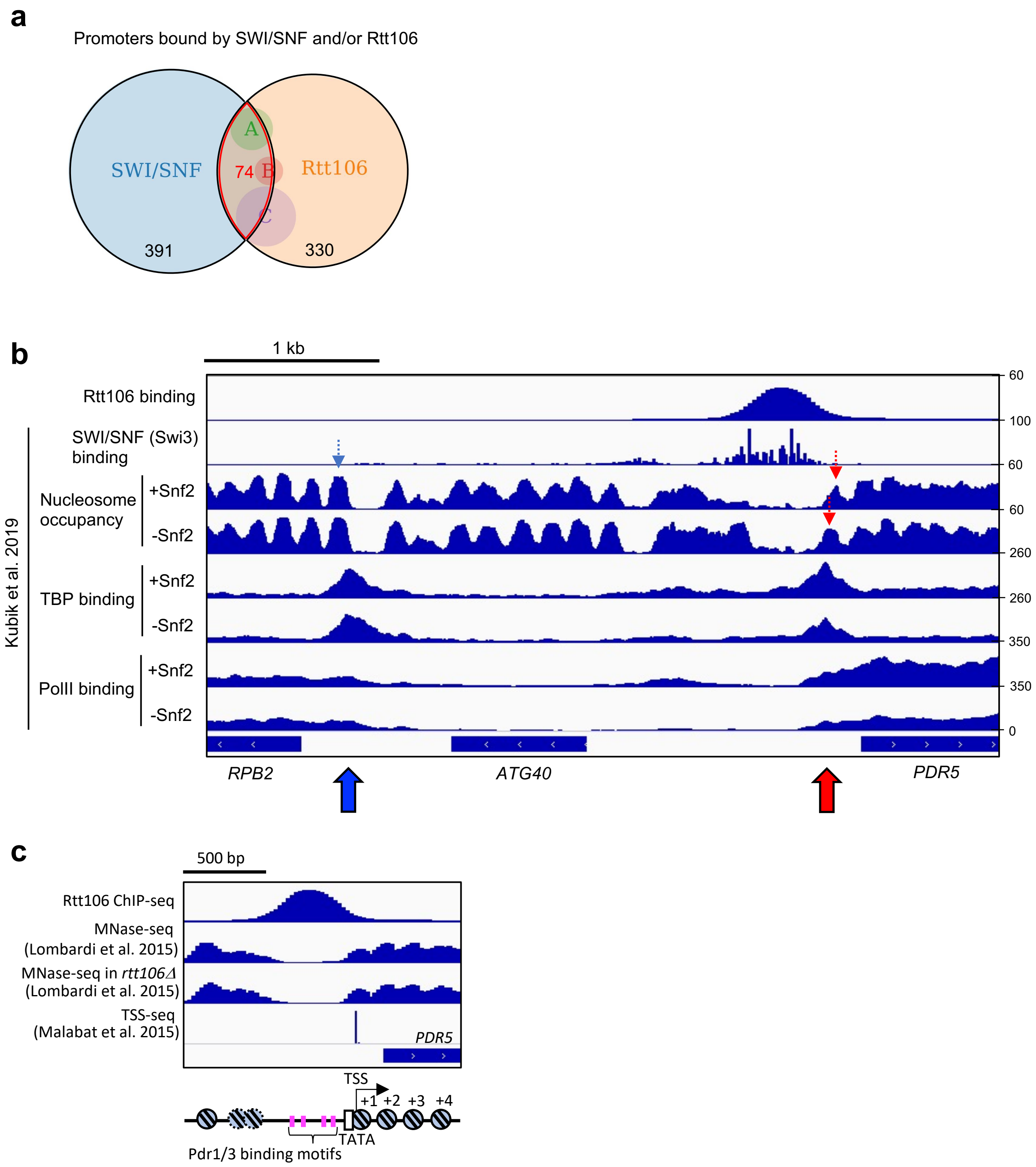

Supplementary Figure 7. Nucleosome positioning and occupancy at the *PDR5* locus in the presence and absence of Snf2 and Rtt106, and the number of promoters bound by SWI/SNF and/or Rtt106.
(a) Venn chart showing the number of promoters bound by either SWI/SNF or Rtt106 (black numbers) or by both (red). A, B, and C indicate Type A-C promoters as in Fig. 6b. (b) Nucleosome positioning and occupancy and bindings of TBP and PolII at the *RPB2-ATG40-PDR5* locus, along with bindings of Swi3 and Rtt106. Nucleosome positioning data (MNase-seq), bindings of TBP and PolII (ChIP-seq) and binding of Swi3 (ChEC-seq) at the *RPB2-ATG40-PDR5* locus extracted from published datasets in Kubik et al. 2019 (ref. 5). At the *PDR5* promoter, the +1 nucleosome was shifted in the absence of Snf2 (dashed red arrow) and bindings of TBP and PolII were decreased in the absence of Snf2 (thick red arrow), while at the *RPB2* promoter depletion of Snf2 caused no change in the position of the +1 nucleosome (dashed blue arrow) or bindings of TBP and PolII (thick blue arrow). (c) Nucleosome positioning and occupancy at the *PDR5* promoter in the presence and absence of Rtt106. Data of nucleosome positioning (MNase-seq) at the *PDR5* locus were extracted from the published datasets in Lombardi et al. 2015 (ref. 1). TSS-seq extracted from Malabat et al. 2015 (ref. 6).

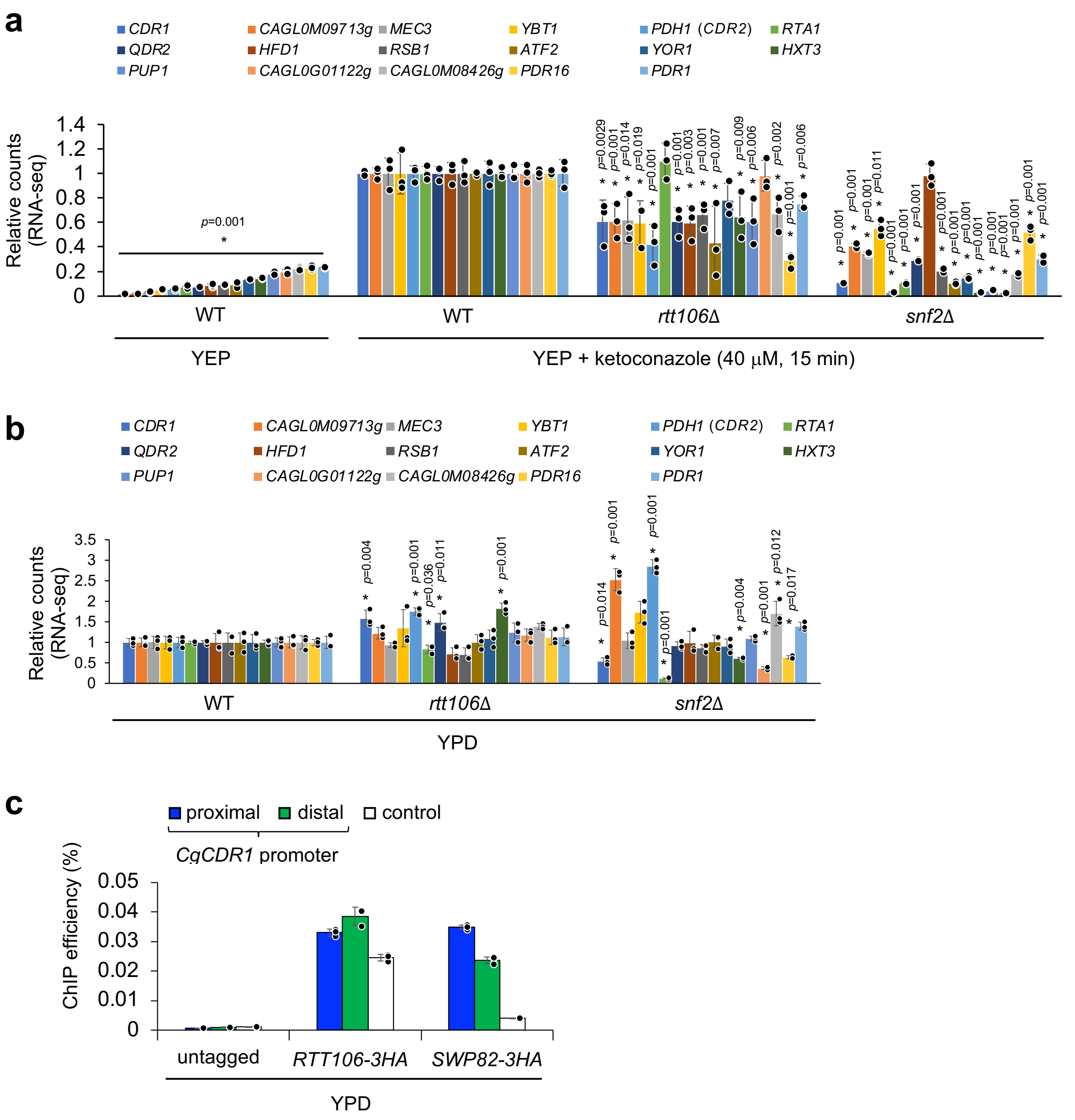

Supplementary Figure 8. RNA-seq and ChIP-qPCR analyses of *C. glabrata* strains.

(a) Normalised read counts of transcripts from genes which can be induced by ketoconazole. Read counts are shown relative to WT (the *C. glabrata* reference strain ATCC 2001) in cells treated with ketoconazole in YEP were shown. All genes whose transcripts increased more than 4-fold on ketoconazole treatment in WT are shown. Data are presented as mean values +/- SD (n = 3 biological replicates). Asterisks indicate genes whose expression is significantly lower (P<0.05, one-way ANOVA with post-hoc Tukey HSD tests), compared to that in WT treated with ketoconazole. (b) Read counts of transcripts prepared from *C. glabrata* lacking CgRtt106 or CgSnf2 normalised to those prepared from WT in YPD. Data are presented as mean values +/- SD (n = 3 biological replicates). Asterisks indicate genes whose expression is significantly altered (P<0.05, one-way ANOVA with post-hoc Tukey HSD tests), compared to WT in YPD. (c) ChIP-qPCR analyses of CgRtt106 and the CgSwp82 SWI/SNF subunit at the *CgCDR1* promoter in YPD (blue, proximal; green, distal, as illustrated in Figure 7b. Data are presented as mean values of 3 technical replicates +/- SD (n = 1 biological experiment). No statistical analysis was carried out. Control (white), a coding region of the *CgADY3* gene (a meiosis gene not expressed in YPD).

Supplementary Table 1. Changes of mRNA level of PDR genes in absence of Rtt106 and Pdr3 in YPD analysed by RNA-seq

gene name	Rtt106 binding to promoter (analysed by ChIP-seq in Fig. 2)	*rtt106Δ*/WT (log2)	*pdr3Δ rtt106Δ*/WT (log2)
PDR5	yes	-2.0516	-2.7227
SNQ2	yes	-0.4707	-0.6926
PDR15	yes	-0.9578	-1.7397
PDR3	yes	-0.7805	-13.4112
YGR035C	yes	-0.8245	-1.7794
IPT1	yes	-0.7381	-0.6557
VHR1	yes	-0.5398	-0.6679
RSB1	yes	-1.9445	-2.3552
YMR102C	yes	-1.0427	-0.5265
RTS3	yes	0.0319	-0.1863
YGR161W-C	yes	-0.5694	-0.9480
LAC1	yes	-0.2934	-0.2221
CIS1	yes	-3.1423	-3.7244
ICY1	yes	-0.7833	-1.5518
SPO24	yes	-0.6355	-0.6444
PDR16	yes	-0.2940	-0.4793
HXT3	yes	-0.9574	-0.6058
MIG2	yes	-1.6336	-1.2240
CAP1	yes	0.0184	-0.2138
YOR1	no	-0.0901	-0.4117
GRE2	no	0.2577	-0.2001
YPL088W	no	0.3451	0.2082
ICT1	no	-0.0658	-0.0294
BDH2	no	-0.4074	-0.6379
YHR139C-A	no	#N/A	#N/A
YHR140W	no	0.2227	0.0098
YGP1	no	0.3295	-0.4685
IML2	no	-0.0137	-0.2477
PDR10	N/A	0.2493	0.2852
YKL071W	N/A	0.4667	0.4934

Supplementary Table 2. Changes of mRNA levels of genes in Types A, B and C in the absence of Rtt106 and Snf2 in YPD, analysed by RNA-seq

Genes whose promoters bound by both SWI/SNF and Rtt106	Cluster in this study (Fig. 2)	Feature (Fig. 2)	Promoter Type (Fig. 2)	*rtt106Δ*/WT (log2)	*snf2Δ*/WT (log2)
FAA4	1	broad	A	-0.821	0.504
SNQ2	1	PDRE	A	-0.471	-0.580
PDR3	1	PDRE	A	-0.780	-1.227
CIS1	1	PDRE	A	-3.142	-2.346
LAC1	1	PDRE	A	-0.293	-0.060
YMR102C	1	PDRE	A	-1.043	-1.059
PDR5	1	PDRE	A	-2.052	-2.419
YGR161W-C	1	PDRE	A	-0.569	1.115
IPT1	1	PDRE	A	-0.738	-0.041
RSB1	1	PDRE	A	-1.944	-5.701
RTS3	1	PDRE	A	0.032	1.786
ICY1	1	PDRE	A	-0.783	0.186
YGR035C	1	PDRE	A	-0.824	-4.546
VHR1	1	PDRE	A	-0.540	-0.325
SPO24	1	PDRE	A	-0.635	0.642
PDR15	1	PDRE	A	-0.958	-1.558
HTB1	2	histone	B	-0.339	-0.243
HHF2	2	histone	B	-0.610	0.295
HTA1	2	histone	B	0.320	-0.863
ECL1	2	peak	B	-0.551	1.573
AGP1	2	peak	B	0.356	-0.716
ALD6	2	PDRE	B	-1.626	-0.173
FTR1	7	peak	C	-0.105	0.009
CIN1	7	peak	C	0.189	0.762
UTH1	7	peak	C	-0.145	-0.127
AMN1	7	peak	C	-0.960	-0.301
SOK2	7	peak	C	-0.122	-0.152
PUT4	7	peak	C	1.204	3.670
HAP4	7	peak	C	-1.023	-0.592
IES6	8	peak	C	0.017	0.486
AAC1	8	peak	C	0.176	2.427
MDH2	8	peak	C	-1.246	-0.725
SNA2	8	peak	C	-0.254	1.331
YEL007W	8	peak	C	#N/A	#N/A
CWP2	8	peak	C	0.174	-0.074
FUI1	8	peak	C	-2.212	-1.514
WSC4	8	peak	C	0.289	-1.625
GLY1	8	peak	C	-0.350	0.094

Supplementary Table 3. Yeast strains used in this study

Name	Relevant genotype	Reference
BY4741	*MATa his3Δ1 leu2Δ0 met15Δ0 ura3Δ0*	Brachmann et al. 1998 (ref. 7)
BY4742	*MATalpha his3Δ1 leu2Δ0 lys2Δ0 ura3Δ0*	Brachmann et al. 1998 (ref. 7)
TKY147	BY4741 *RTT106-6HA::hphNT1*	Gali et al., 2018 (ref. 8)
VNY22	BY4741 *RTT106-6HA::hphNT1 pdr1Δ::kanMX pdr3Δ::natMX*	this study
VNY27	BY4741 *RTT106-6HA::hphNT1 hir1Δ::kanMX*	this study
VNY31	BY4741 *RTT106-6HA::hphNT1 yta7Δ::kanMX*	this study
VNY2.1	BY4741 *rtt106Δ::kanMX*	this study
VNY45	BY4741 *pdr3Δ::natMX*	this study
VNY49	BY4741 *pdr3Δ::natMX rtt106Δ::kanMX*	this study
VNY70	BY4741 *pdr1Δ::natNT2*	this study
VNY71	BY4741 *pdr1Δ::natNT2 rtt106Δ::kanMX*	this study
BRY3	BY4741 *PDR5-3HA::HIS3MX*	this study
BRY4	BY4741 *PDR5-3HA::HIS3MX rtt106Δ::kanMX*	this study
pdr5Δ	BY4741 *pdr5Δ::kanMX*	EUROSCARF deletion collection
VNY50	BY4741 *pdr1Δ::kanMX pdr3Δ::natMX*	this study
VNY34	BY4741 *RTT106-6HA::hphNT1 pdr1Δ::kanMX*	this study
VNY24	BY4741 *RTT106-6HA::hphNT1 pdr3Δ::natMX*	this study
VNY30	BY4741 *RTT106-6HA::hphNT1 asf1Δ::kanMX*	this study
VNY33	BY4741 *RTT106-6HA::hphNT1 rtt109Δ::kanMX*	this study
VNY66	BY4741 *RTT106-6HA::hphNT1 3FLAG-PDR3*	this study
TKY583	BY4741 *PDR3-3FLAG::natMX*	this study
TKY584	BY4741 *PDR3-6HA::hphNT1*	this study
TKY585	BY4741 *PDR3-13Myc::HIS3MX*	this study
TKY586	BY4741 *3HA-PDR3*	this study
TKY587	BY4741 *2HA-PDR3*	this study
TKY588	BY4741 *3Myc-PDR3*	this study
TKY589	BY4741 *PDR3-13Myc::HIS3MX rtt106Δ::kanMX*	this study
TKY590	BY4741 *PDR3-13Myc::HIS3MX snf2Δ::kanMX*	this study
TKY591	BY4741 *PDR3-13Myc::HIS3MX asf1Δ::kanMX*	this study
TKY545	BY4741 *pdr1-3*	this study
TKY547	BY4741 *pdr3-2*	this study
TKY549	BY4741 *pdr1-3 rtt106Δ::kanMX*	this study
TKY551	BY4741 *pdr3-2 rtt106Δ::kanMX*	this study
TKY553	BY4741 *pdr1-3 snf6Δ::kanMX*	this study
TKY557	BY4741 *pdr3-2 snf6Δ::kanMX*	this study
TKY493	*MAT***a** *arg4Δ::natMX4 his3Δ1 leu2Δ0 lys2Δ0 ura3Δ0 trp1Δ::CMV-LacI-3FLAG::URA3*	this study
TKY494	*MAT***a** *arg4Δ::natMX4 his3Δ1 leu2Δ0 lys2Δ0 ura3Δ0 trp1Δ::CMV-LacI-3FLAG::URA3 RTT106-6HA::hphNT1*	this study
VNY40	BY4741 *SWP82-6HA::hphNT1*	this study
VNY62	BY4741 *SWP82-6HA::hphNT1 pdr1Δ::kanMX pdr3Δ::natMX*	this study
TKY507	BY4741 *SWP82-6HA::hphNT1 pdr1Δ::kanMX*	this study
TKY508	BY4741 *SWP82-6HA::hphNT1 pdr3Δ::natMX*	this study
TKY541	*MATa ura3-52 trp1-289 leu2-3,112 prb1-1122 prc1-407 pep4-3 SNF6-3FLAG::kanMX*	this study
snf2Δ	BY4741 *snf2Δ::kanMX*	EUROSCARF deletion collection
snf5Δ	BY4741 *snf5Δ::kanMX*	EUROSCARF deletion collection
swi3Δ	BY4741 *swi3Δ::kanMX*	EUROSCARF deletion collection
snf6Δ	BY4741 *snf6Δ::kanMX*	EUROSCARF deletion collection
snf12Δ	BY4742 *snf12Δ::kanMX*	EUROSCARF deletion collection
snf11Δ	BY4741 *snf11Δ::kanMX*	EUROSCARF deletion collection
swp82Δ	BY4741 *swp82Δ::kanMX*	EUROSCARF deletion collection
CBS138	*Candida glabrata* wild-type (ATCC 2001)	Dujon et al. 2004 (ref. 9)
VNG3	CBS138 *Cgrtt106Δ::NAT*	this study
TKG1	CBS138 *Cgsnf2Δ::NAT*	this study
TKG3	CBS138 *CgRTT106-3HA::NAT*	this study
TKG5	CBS138 *CgSWP82-3HA::NAT*	this study
VNG5	CBS138 *Cgcdr1Δ::NAT*	this study

Supplementary References

1. Lombardi, L. M., Davis, M. D. & Rine, J. Maintenance of nucleosomal balance in cis by conserved AAA-ATPase Yta7. Genetics 199, 105-116, doi:10.1534/genetics.114.168039 (2015).

2. Kapoor, P. et al. Regulation of Mec1 kinase activity by the SWI/SNF chromatin remodeling complex. Genes Dev 29, 591-602, doi:10.1101/gad.257626.114 (2015).

3. Deshpande, I. et al. Structural Basis of Mec1-Ddc2-RPA Assembly and Activation on Single-Stranded DNA at Sites of Damage. Mol Cell 68, 431-445 e435, doi:10.1016/j.molcel.2017.09.019 (2017).

4. Kemmeren, P. et al. Large-scale genetic perturbations reveal regulatory networks and an abundance of gene-specific repressors. Cell 157, 740-752, doi:10.1016/j.cell.2014.02.054 (2014).

5. Kubik, S. et al. Opposing chromatin remodelers control transcription initiation frequency and start site selection. Nat Struct Mol Biol 26, 744-754, doi:10.1038/s41594-019-0273-3 (2019).

6. Malabat, C., Feuerbach, F., Ma, L., Saveanu, C. & Jacquier, A. Quality control of transcription start site selection by nonsense-mediated-mRNA decay. Elife 4, e06722, doi:10.7554/eLife.06722 (2015).

7. Brachmann, C. B. et al. Designer deletion strains derived from Saccharomyces cerevisiae S288C: a useful set of strains and plasmids for PCR-mediated gene disruption and other applications. Yeast (Chichester, England) 14, 115-132 (1998).

8. Gali, V. K. et al. Identification of Elg1 interaction partners and effects on post-replication chromatin re-formation. PLoS Genet 14, e1007783, doi:10.1371/journal.pgen.1007783 (2018).

9. Dujon, B. et al. Genome evolution in yeasts. Nature 430, 35-44, doi:10.1038/nature02579 (2004).
