# Supplementary material for: SWI/SNF and the histone chaperone Rtt106 drive expression of the Pleiotropic Drug Resistance network genes
Source: Nat Commun. 2022 Apr 12;13:1968. doi: 10.1038/s41467-022-29591-z (PMC9005695; doi:10.1038/s41467-022-29591-z)
Supplement: Supplementary file 3 — Description of additional Supplementary File [file 41467_2022_29591_MOESM3_ESM.pdf]

Descriptions of additional Supplementary Data files

Supplementary Data 1
ChIP-seq analysis of Rtt106 showing its binding to gene promoters in WT and its change in *pdr1*Δ *pdr3*Δ, *hir1*Δ, and *yta7*Δ. Colour codes are used as defined in Fig. 2e. Clusters and Promoter types are defined as in Figs. 2d and 2e.

Supplementary Data 2
Rtt106-bound promoters showing regulated expression either in response to environmental changes or during the cell cycle.

Supplementary Data 3
RNA-seq data showing changes in mRNA level in *rtt106*Δ, *pdr1*Δ and *pdr3*Δ compared to WT grown in YPD. FDR, false discovery rate; RPKM, reads per kilobase of transcript per million mapped reads.

Supplementary Data 4
List of published papers examining *PDR5* expression dependence on Pdr1 and Pdr3.

Supplementary Data 5
Proteins identified and quantified (by at least 5 ratio counts) by SILAC-based quantitative proteomic analysis of isolated minichromosomes. Data were analysed by MaxQuant software as described in Methods.

Supplementary Data 6
Gene promoters bound by SWI/SNF and Rtt106. Promoters bound by SWI/SNF are defined based on Swi3 binding categorised into Clusters I and VI in Kubik et al. 2019. Promoters designated as Types A, B and C in this study are defined as Rtt106-bound promoters. Colour codes were used as in Fig. 6b.

Supplementary Data 7
Oligonucleotides used in this study.
