# Supplementary material for: SWI/SNF and the histone chaperone Rtt106 drive expression of the Pleiotropic Drug Resistance network genes
Source: Nat Commun. 2022 Apr 12;13:1968. doi: 10.1038/s41467-022-29591-z (PMC9005695; doi:10.1038/s41467-022-29591-z)
Supplement: Supplementary file 11 — Reporting Summary [file 41467_2022_29591_MOESM11_ESM.pdf]

nature research

Corresponding author(s): Takashi Kubota

Last updated by author(s): Feb 26, 2022

Reporting Summary

Nature Research wishes to improve the reproducibility of the work that we publish. This form provides structure for consistency and transparency in reporting. For further information on Nature Research policies, see our Editorial Policies and the Editorial Policy Checklist.

Statistics

For all statistical analyses, confirm that the following items are present in the figure legend, table legend, main text, or Methods section.

n/a	Confirmed	
☐	☒	The exact sample size (*n*) for each experimental group/condition, given as a discrete number and unit of measurement
☐	☒	A statement on whether measurements were taken from distinct samples or whether the same sample was measured repeatedly
☐	☒	The statistical test(s) used AND whether they are one- or two-sided<br>*Only common tests should be described solely by name; describe more complex techniques in the Methods section.*
☒	☐	A description of all covariates tested
☒	☐	A description of any assumptions or corrections, such as tests of normality and adjustment for multiple comparisons
☐	☒	A full description of the statistical parameters including central tendency (e.g. means) or other basic estimates (e.g. regression coefficient) AND variation (e.g. standard deviation) or associated estimates of uncertainty (e.g. confidence intervals)
☐	☒	For null hypothesis testing, the test statistic (e.g. *F*, *t*, *r*) with confidence intervals, effect sizes, degrees of freedom and *P* value noted<br>*Give P values as exact values whenever suitable.*
☒	☐	For Bayesian analysis, information on the choice of priors and Markov chain Monte Carlo settings
☒	☐	For hierarchical and complex designs, identification of the appropriate level for tests and full reporting of outcomes
☒	☐	Estimates of effect sizes (e.g. Cohen's *d*, Pearson's *r*), indicating how they were calculated

Our web collection on statistics for biologists contains articles on many of the points above.

Software and code

Policy information about availability of computer code

Data collection	No software was used.

Data analysis

We used the following software or web interface for data analysis:
The Galaxy web interface (https://usegalaxy.org)
TrimGalore! (0.6.4)
HISAT2 (2.1.0)
SAMtools (1.9)
featureCounts (part of the sub read version 1.6.2 package)
edgeR (3.26.8)
Gene Ontology Term Finder (https://www.yeastgenome.org/goTermFinder) (vesion 0.86)
MaxQaunt (1.6.5.0)
Proteome Discoverer (2.2.0.388)
BWA(0.7.17.4)
DeepTools bamCoverage (3.1.2.0.0)
DeepTools bamCompare (3.1.2.0.0)
DeepTools computeMatrix (3.1.2.0.0)
DeepTools plotHeatmap (3.1.2.0.1)
Integrated Genome Browser (2.4.16)

The images of blots and gels were obtained and analysed in Image Lab (5.2.1, Bio-Rad). Mean and standard deviations (SDs) from numerical data was calculated and tabulated using Microsoft Excel 2019 (version 16.54).

For manuscripts utilizing custom algorithms or software that are central to the research but not yet described in published literature, software must be made available to editors and reviewers. We strongly encourage code deposition in a community repository (e.g. GitHub). See the Nature Research guidelines for submitting code & software for further information.

Data

Policy information about availability of data

All manuscripts must include a data availability statement. This statement should provide the following information, where applicable:

- Accession codes, unique identifiers, or web links for publicly available datasets
- A list of figures that have associated raw data
- A description of any restrictions on data availability

All raw-data files for ChIP-seq and RNA-seq data were uploaded to ArrayExpress under accession numbers: E-MTAB-9787 (https://www.ebi.ac.uk/arrayexpress/experiments/E-MTAB-9787/) and E-MTAB-11013 (https://www.ebi.ac.uk/arrayexpress/experiments/E-MTAB-11013/).

The mass spectrometry proteomics data have been deposited to the ProteomeXchange Consortium via the PRIDE partner repository with the dataset identifier PXD028798 (https://www.ebi.ac.uk/pride/archive/projects/PXD028798). Source data are provided with this paper.

Publicly available datasets used in this study are:
GSM3452517 (https://www.ncbi.nlm.nih.gov/geo/query/acc.cgi?acc=GSM3452517) (Swi3 ChEC-seq),
GSM3177770 (https://www.ncbi.nlm.nih.gov/geo/query/acc.cgi?acc=GSM3177770) (MNase-seq, +Snf2),
GSM3177771 (https://www.ncbi.nlm.nih.gov/geo/query/acc.cgi?acc=GSM3177771) (MNase-seq, -Snf2),
GSM3452556 (https://www.ncbi.nlm.nih.gov/geo/query/acc.cgi?acc=GSM3452556) (TBP ChIP-seq, +Snf2),
GSM3452557 (https://www.ncbi.nlm.nih.gov/geo/query/acc.cgi?acc=GSM3452557) (TBP ChIP-seq, -Snf2),
SRX648019 (https://www.ncbi.nlm.nih.gov/sra/SRX648019[accn]) (MNase-seq, WT),
SRX648516 (https://www.ncbi.nlm.nih.gov/sra/SRX648516[accn]) (MNase-seq, rtt106 deletion mutant),
SRX648532 (https://www.ncbi.nlm.nih.gov/sra/SRX648532[accn]) (Yta7 ChIP-seq),
GSM1565066 (https://www.ncbi.nlm.nih.gov/geo/query/acc.cgi?acc=GSM1565066) (TSS-seq),
Saccharomyces cerevisiae genome (sacCer3, April 2011) (https://hgdownload.soe.ucsc.edu/goldenPath/sacCer3/chromosomes/),
Saccharomyces cerevisiae protein database (UP000002311_559292) (https://www.uniprot.org/proteomes/UP000002311).

Field-specific reporting

Please select the one below that is the best fit for your research. If you are not sure, read the appropriate sections before making your selection.

☒ Life sciences ☐ Behavioural & social sciences ☐ Ecological, evolutionary & environmental sciences

For a reference copy of the document with all sections, see nature.com/documents/nr-reporting-summary-flat.pdf

Life sciences study design

All studies must disclose on these points even when the disclosure is negative.

Sample size

All relevant sample sizes are described in the legend to each figure and/or in the Methods section. No calculations were performed to determine sample size. Statistical tests were not used to predetermine sample size. Sample size was determined based on standards for experimental biology studies, attempting to have a minimum of n = 3 biological replicates with sufficient reproducibility. Sample sizes for ChIP-seq and RNA-seq were determined based on ENCODE Experiment Guidelines (https://www.encodeproject.org/data-standards/). We

performed two replicates per condition for each ChIP-seq experiment, and three replicates per condition for each RNA-seq experiment. Mass spectrometry analysis for isolated minichromosomes was performed once, and therefore only proteins quantified by at least 5 ratio counts were presented, allowing reliable quantification of proteins in the sample analysed. Subsequently, binding of SWI/SNF (identified by mass spectrometry analysis of minichromosome isolation) to the PDR5 promoter was confirmed by ChIP-qPCR with three biological replicates.

Data exclusions

No data were excluded from analysis.

Replication

We performed three replicates per condition for each RNA-seq experiment (and observed similar results) and two replicates per condition for each ChIP-seq experiment. Two ChIP-seq data show high correlation coefficient analysed by the plotCorrelation tool in the Galaxy web interface.
For Northern blot and GST pull-down analyses and testing protein level of Pdr5 (Fig. 4b), each experiment was repeated three times independently with similar results
The experiments for confirming expression of a series of epitope-tagged Pdr3 (supplementary figures 3b and 3c) were performed once with at least 2 different isolates (which showed similar results).
Minichromosome isolation and purification of the SWI/SNF complex followed by SYPRO Ruby staining (supplementary figures 6a and 6b) were repeated three times independently with similar results.
Enrichment of the SWI/SNF complex by immunopurification was performed three times and assessed by comparing the band pattern on the gel with that in published data (n = 3 biological replicates, with similar results).

Randomization

Biological samples were allocated into groups based on the conditions used.

Blinding

All experiments did not employ blinding since readouts were quantitative and not prone to subjective judgment of investigators.

Reporting for specific materials, systems and methods

We require information from authors about some types of materials, experimental systems and methods used in many studies. Here, indicate whether each material, system or method listed is relevant to your study. If you are not sure if a list item applies to your research, read the appropriate section before selecting a response.

Materials & experimental systems

n/a	Involved in the study	
☐	☒	Antibodies
☒	☐	Eukaryotic cell lines
☒	☐	Palaeontology and archaeology
☒	☐	Animals and other organisms
☒	☐	Human research participants
☒	☐	Clinical data
☒	☐	Dual use research of concern

Methods

n/a	Involved in the study	
☐	☒	ChIP-seq
☒	☐	Flow cytometry
☒	☐	MRI-based neuroimaging

Antibodies

Antibodies used

Western blot primary antibodies:
mouse monoclonal anti-HA antibody (1:5000, Covance or Biolegend, HA.11, clone 16B12)
mouse monoclonal anti-FLAG M2 antibody (1:5000, Sigma or Merck, F1804, clone M2)
mouse monoclonal anti-FLAG M2-HRP (1:5000, Merck, A8592, clone M2)
rabbit polyclonal anti-myc antibody (1:50,000, abcam, ab9106)
mouse monoclonal anti-His antibody (1:5000, Biolegend, 652502, clone J099B12)

Western blot secondary antibodies:
goat anti-mouse IgG (H+L) antibody conjugated with horseradish peroxidase (HRP) (1:15,000, Bio-Rad, 1706516)
goat anti-rabbit IgG (H+L) antibody conjugated with horseradish peroxidase (HRP) (1:15,000, Bio-Rad, 1706515)

Antibodies used for ChIP and immunoprecipitation:
mouse monoclonal anti-HA antibody (5 micrograms per ChIP sample, Covance or Biolegend, HA.11, clone 16B12)
mouse monoclonal anti-myc antibody (5 micrograms per ChIP sample, MBL, M047-3, clone PL14)
mouse monoclonal anti-FLAG M2 antibody (30 micrograms per immunopurification sample, Sigma or Merck, F1804, clone M2)

Validation

mouse monoclonal anti-HA antibody (Covance or Biolegend, HA.11, clone 16B12): one of the most commonly used antibodies against HA epitope tag, e.g., in Gali VK, Dickerson D, Katou Y, Fujiki K, Shirahige K, et al. (2018) Identification of Elg1 interaction partners and effects on post-replication chromatin re-formation. PLOS Genetics 14(11): e1007783

mouse monoclonal anti-FLAG antibody (Sigma or Merck, F1804, clone M2): one of the most commonly used antibodies against FLAG epitope tag, e.g.,Gali VK, Dickerson D, Katou Y, Fujiki K, Shirahige K, et al. (2018) Identification of Elg1 interaction partners and effects on post-replication chromatin re-formation. PLOS Genetics 14(11): e1007783

mouse monoclonal anti-Myc antibody (MBL, M047-3, clone PL14): one of the commonly used antibodies against Myc epitope tag,

e.g., 3. Paul Solomon Devakumar, LJ, Gaubitz, C, Lundblad, V, Kelch, BA, and Kubota, T. (2019) Effective mismatch repair depends on timely control of PCNA retention on DNA by the Elg1 complex. Nucleic Acids Research 47: 6826-41.

mouse monoclonal anti-His antibody (Biolegend, 652502, clone J099B12): validated in https://www.biolegend.com/en-us/search-results/purified-anti-his-tag-antibody-7841

rabbit polyclonal anti-myc antibody (abcam, ab9106): one of the commonly used antibodies against Myc epitope tag, https://www.abcam.com/myc-tag-antibody-ab9106.html

ChIP-seq

Data deposition

☒ Confirm that both raw and final processed data have been deposited in a public database such as GEO.

☒ Confirm that you have deposited or provided access to graph files (e.g. BED files) for the called peaks.

Data access links
May remain private before publication.

We deposited raw data to ArrayExpress under accession number: E-MTAB-9787 (http://www.ebi.ac.uk/arrayexpress/experiments/E-MTAB-9787).

Files in database submission

ChIP-seq data (raw and processed data):
TKY147#1 VK7-1 input
VNY22#1 VN2-1 input
VNY27#1 VN7-1 input
VNY31#1 VN3-1 input
TKY147#1 VK7-1 ChIP
VNY22#1 VN2-1 ChIP
VNY27#1 VN7-1 ChIP
VNY31#1 VN3-1 ChIP
TKY147#2 VK7-2 input
VNY22#2 VN2-2 input
VNY27#2 VN7-2 input
VNY31#2 VN3-2 input
TKY147#2 VK7-2 ChIP
VNY22#2 VN2-2 ChIP
VNY27#2 VN7-2 ChIP
VNY31#2 VN3-2 ChIP

Genome browser session
(e.g. UCSC)

We used IGV (The Integrative Genomics Viewer)

Methodology

Replicates

We performed two replicates per condition for each ChIP-seq experiment.

Sequencing depth

single-end 75-bp reads. ~15M reads per sample.

Antibodies

mouse monoclonal anti-HA antibody (HA.11 clone 16B12, Covance)

Peak calling parameters

To calculate Rtt106 enrichment or occupancy at each promoter region, enrichment of sequence reads in ChIP samples over corresponding Input samples was first calculated using DeepTools bamCompare, with bin size 50 bp. To extract values around the TSS of each gene, heatmap was then plotted using annotation of TSS, DeepTools computeMatrix and plotHeatmap. Finally, the data table underlying the plots was exported and utilised for calculating Rtt106 enrichment in a region spanning 500 bp upstream of the TSS of each gene. To calculate fold change of Rtt106 signals in mutants over WT at each promoter, enrichment of ChIP sequence reads in those mutants over WT was calculated.

Data quality

Two ChIP-seq data show high correlation coefficient analysed by the plotCorrelation tool in the Galaxy web interface.

Software

The Galaxy web interface (https://usegalaxy.org)
