# Supplementary material for: SWI/SNF and the histone chaperone Rtt106 drive expression of the Pleiotropic Drug Resistance network genes
Source: Nat Commun. 2022 Apr 12;13:1968. doi: 10.1038/s41467-022-29591-z (PMC9005695; doi:10.1038/s41467-022-29591-z)
Supplement: Supplementary file 12 — Source Data [file 41467_2022_29591_MOESM12_ESM.zip › SourceData_uncropped_gels_blots_final_18Feb22.pptx]

## Slide 1
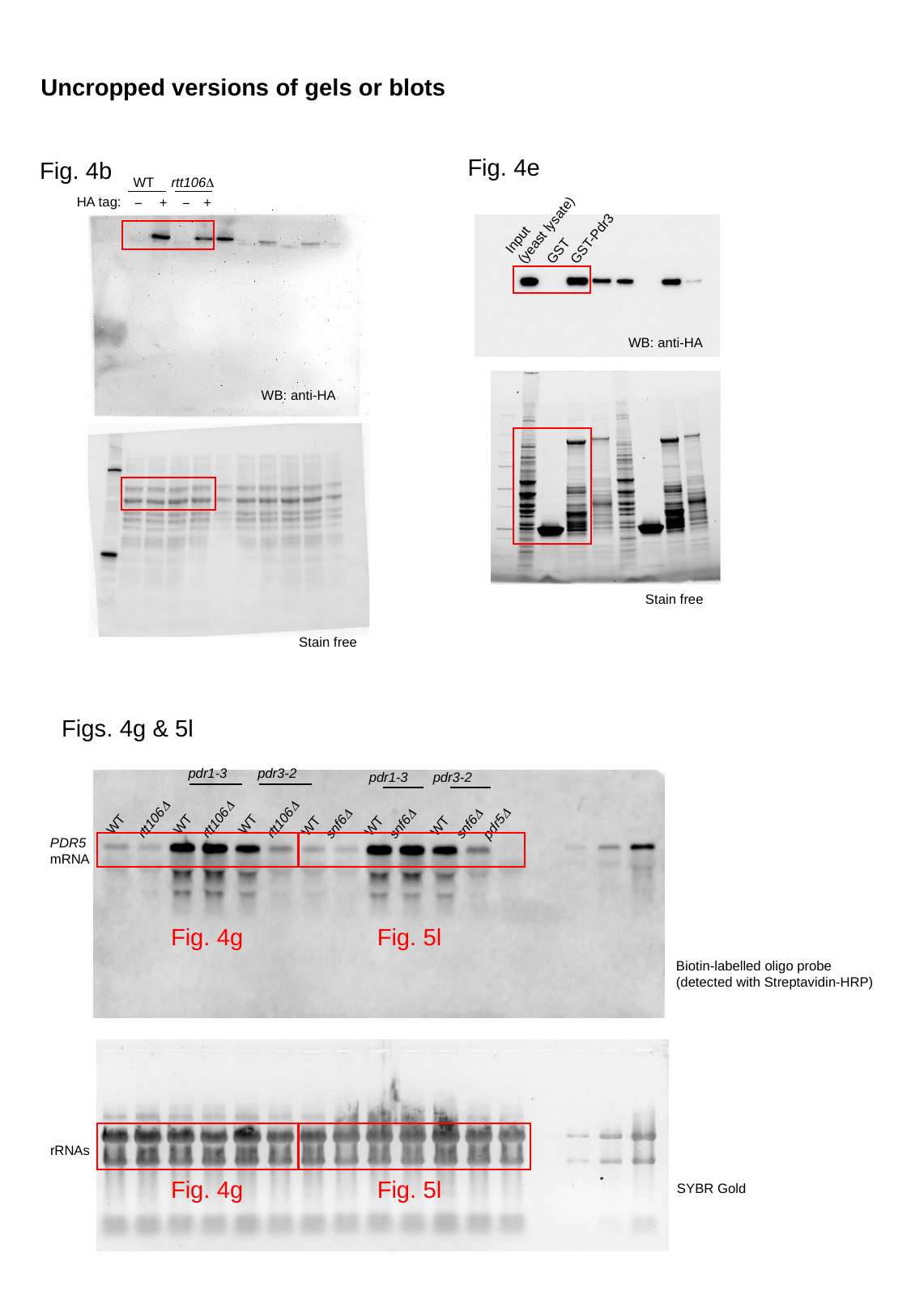

Uncropped versions of gels or blots
Fig. 4e
Fig. 4b
WT
rtt106Δ
HA tag:
+
+
−
−
Input
(yeast lysate)
GST-Pdr3
GST
WB: anti-HA
WB: anti-HA
Stain free
Stain free
Figs. 4g & 5l
pdr1-3
pdr3-2
pdr1-3
pdr3-2
rtt106D
rtt106D
rtt106D
pdr5D
snf6D
snf6D
snf6D
WT
WT
WT
WT
WT
WT
PDR5 mRNA
Fig. 4g
Fig. 5l
Biotin-labelled oligo probe
(detected with Streptavidin-HRP)
rRNAs
Fig. 4g
Fig. 5l
SYBR Gold

## Slide 2
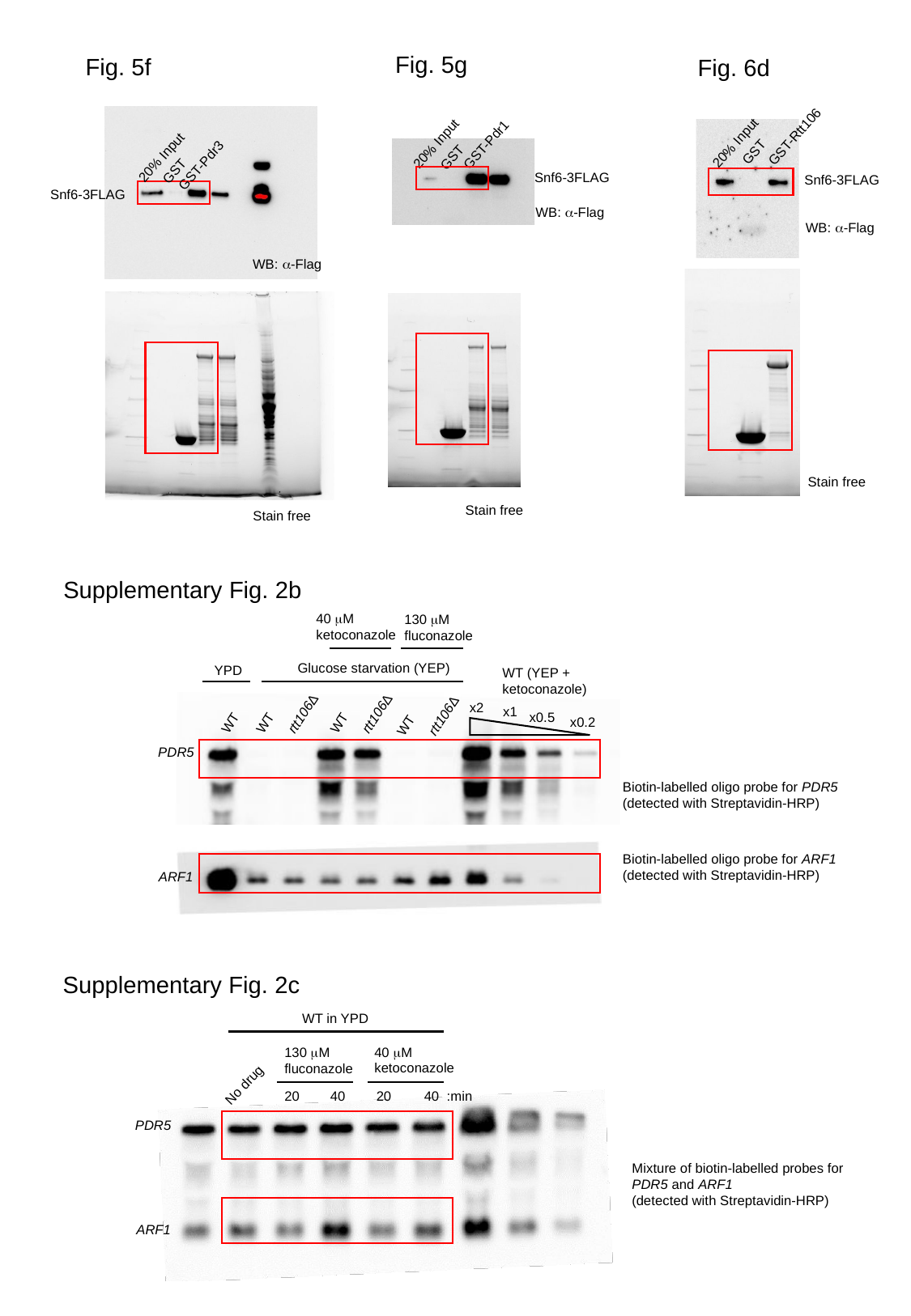

Fig. 5g
Fig. 5f
Fig. 6d
GST-Rtt106
20% Input
20% Input
GST-Pdr1
GST
20% Input
GST
GST-Pdr3
GST
Snf6-3FLAG
Snf6-3FLAG
Snf6-3FLAG
WB: a-Flag
WB: a-Flag
WB: a-Flag
Stain free
Stain free
Stain free
Supplementary Fig. 2b
40 mM
ketoconazole
130 mM fluconazole
Glucose starvation (YEP)
YPD
WT (YEP + ketoconazole)
rtt106Δ
rtt106Δ
x2
rtt106Δ
x1
x0.5
WT
WT
WT
WT
x0.2
PDR5
Biotin-labelled oligo probe for PDR5
(detected with Streptavidin-HRP)
Biotin-labelled oligo probe for ARF1
(detected with Streptavidin-HRP)
ARF1
Supplementary Fig. 2c
WT in YPD
40 mM
ketoconazole
130 mM fluconazole
No drug
20
20
40
40 :min
PDR5
Mixture of biotin-labelled probes for PDR5 and ARF1
(detected with Streptavidin-HRP)
ARF1

## Slide 3
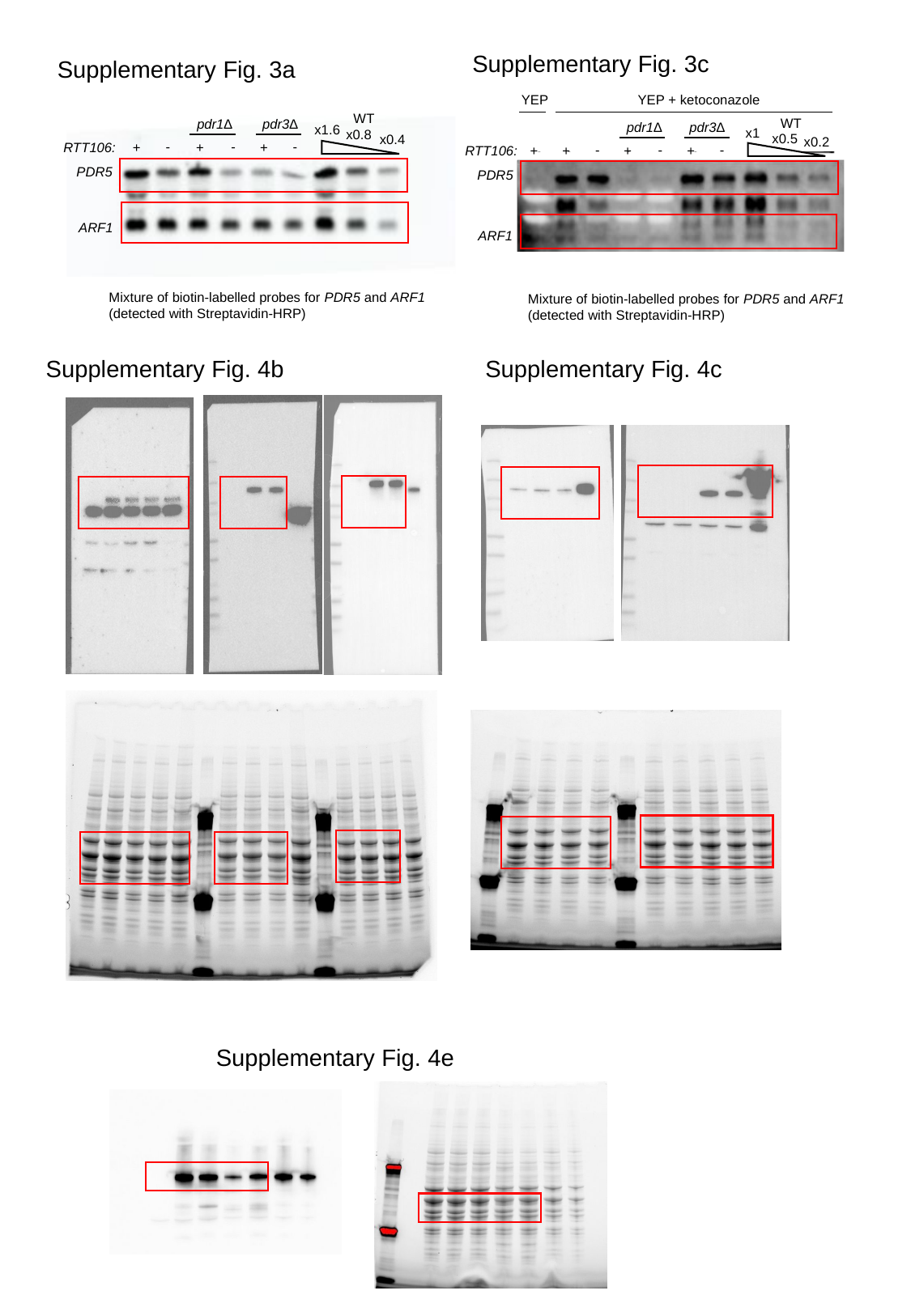

Supplementary Fig. 3c
Supplementary Fig. 3a
YEP
YEP + ketoconazole
WT
WT
pdr1∆
pdr3∆
pdr1∆
pdr3∆
x1.6
x1
x0.8
x0.5
x0.4
x0.2
-
-
-
+
+
+
RTT106:
-
-
-
+
+
+
+
RTT106:
PDR5
PDR5
ARF1
ARF1
Mixture of biotin-labelled probes for PDR5 and ARF1
(detected with Streptavidin-HRP)
Mixture of biotin-labelled probes for PDR5 and ARF1
(detected with Streptavidin-HRP)
Supplementary Fig. 4b
Supplementary Fig. 4c
Supplementary Fig. 4e

## Slide 4
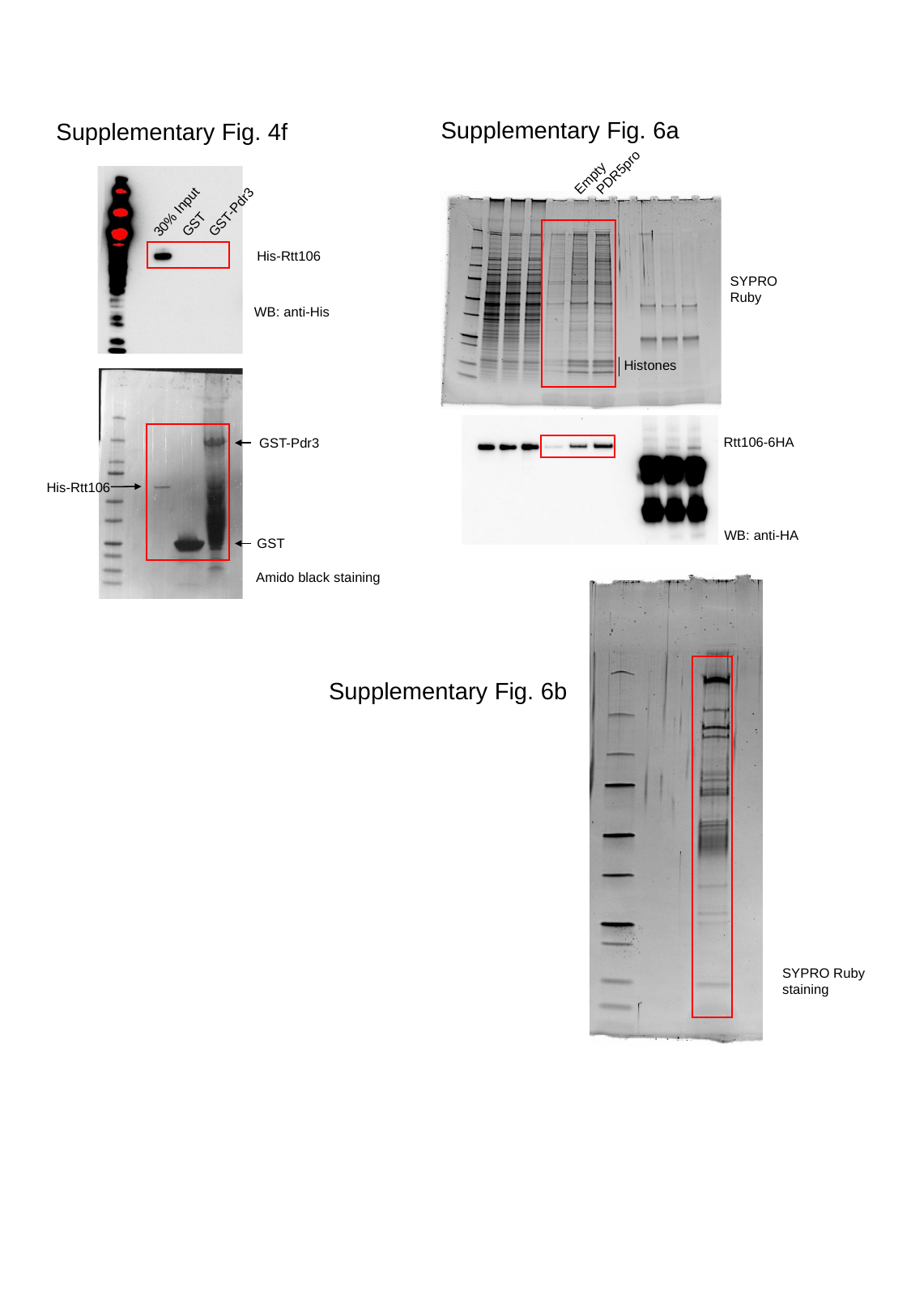

Supplementary Fig. 6a
Supplementary Fig. 4f
PDR5pro
Empty
30% Input
GST-Pdr3
GST
His-Rtt106
SYPRO
Ruby
WB: anti-His
Histones
Rtt106-6HA
GST-Pdr3
His-Rtt106
WB: anti-HA
GST
Amido black staining
Supplementary Fig. 6b
SYPRO Ruby staining
